# Demographic Differences in Periodic Limb Movement Index and Apnea–Hypopnea Index in a Diverse Clinical Cohort

**DOI:** 10.3390/ijerph22101476

**Published:** 2025-09-24

**Authors:** Lourdes M. DelRosso, Harshil Modi, Alec M. Chan-Golston, Prabhvir Sandhu, Viraj Jain, Moon Park

**Affiliations:** 1 Department of Medicine, University of California San Francisco, Fresno, CA 93701, USA; 2 Health Sciences Research Institute, University of California, Merced, CA 95343, USA; achan-golston@ucmerced.edu (A.M.C.-G.); psandhu11@ucmerced.edu (P.S.); 3 Department of Public Health, University of California, Merced, CA 95343, USA; 4 Department of Chemistry and Biochemistry, Loyola Marymount University, Los Angeles, CA 90045, USA; virajjain7@gmail.com

**Keywords:** periodic limb movements, obstructive sleep apnea, sleep disparities, ethnicity, sex differences, age, PLMI, AHI, polysomnography

## Abstract

This study investigated how age, sex, and ethnicity relate to the severity of periodic limb movement index (PLMI) and obstructive sleep apnea (OSA) in a diverse clinical population. A retrospective analysis was conducted on 711 adults who underwent diagnostic polysomnography between June 2022 and July 2024. The mean age was 57.2 years; 55.6% were female, and the sample was ethnically diverse (53.7% White, 31.6% Hispanic, 6.8% Asian, 6.1% Black, 1.8% Other). PLMI was significantly higher in older adults, males, and White participants. AHI was higher in males and peaked in middle-aged adults (44–62 years). Multivariable regression models showed that male sex independently predicted higher PLMI and AHI, while increasing age was associated with higher PLMI only. Black ethnicity was linked to lower PLMI, and Hispanic ethnicity to higher AHI. These findings emphasize the role of demographic factors in the presentation of sleep disorders and support the need for personalized approaches in screening and diagnosis. Recognizing at-risk subgroups may improve early detection and targeted interventions for both PLMS and OSA in diverse populations.

## 1. Introduction

Obstructive sleep apnea (OSA) and periodic limb movement disorder (PLMD) are two common sleep disorders that contribute significantly to disrupted sleep architecture, excessive daytime sleepiness, and increased risk of comorbidities including cardiovascular disorders, including hypertension, stroke, atrial fibrillation, diabetes, and increased cardiovascular morbidity and mortality [1,2,3]. Obstructive sleep apnea is a sleep-related breathing disorder marked by recurrent episodes of upper airway closure during sleep, leading to reduced (hypopnea) or absent (apnea) airflow despite ongoing respiratory effort. The apnea–hypopnea index (AHI) is defined as the average number of apnea and hypopnea events per hour of sleep is the gold standard metric for quantifying OSA severity [4]. According to established clinical criteria, an AHI of ≥5 events/h, with accompanying symptoms such as daytime sleepiness or cardiovascular complications, meets the threshold for diagnosis, while an AHI of ≥15 events/h is diagnostic irrespective of symptoms [4]. Polysomnographic studies have documented that approximately 936 million adults worldwide aged 30–69 have at least mild OSA (AHI ≥ 5), with 425 million meeting criteria for moderate to severe OSA (AHI ≥ 15) [5]. Among older adults (>65 years), pooled prevalence reaches nearly 36% [6]. This global burden highlights OSA’s silent yet pervasive contribution to cardiometabolic disease, impaired daytime functioning, and elevated morbidity.

Periodic limb movements of sleep (PLMSs) are characterized by rhythmic, stereotypical limb movements, predominantly in the lower limbs, occurring at intervals of roughly 20–40 s during sleep [7]. The periodic limb movement index (PLMI), calculated as the number of such movements per hour via electromyography during polysomnography, is the quantitative standard [8]. A PLMI threshold of ≥15/h in adults (≥5/h in children) is required to diagnose PLMD, but only when these movements cause sleep disruption or daytime impairment [9]. Epidemiological data indicate that PLMD affects approximately 4–11% of adults and 5–8% of children globally, placing patients at risk of the consequences, including poor sleep quality, daytime impairment, and comorbidities [10].

The AHI and PLMI are the primary physiological markers used to quantify the severity of OSA and PLMD, respectively [11,12]. While both conditions are prevalent in the general population, there is substantial variability in their expression based on demographic characteristics such as age, sex, and ethnicity [13,14]. Population studies, such as the Wisconsin Sleep Cohort, have shown that OSA affects 24% of men and 9% of women [15]. Another large population study linked the presence of OSA to cardiovascular risk, including stroke and myocardial infarction [16], providing some insightful information about variations in symptom burden. Black individuals presented with more sleepiness and snoring despite similar AHI [17]. Ethnic differences in presentation were further assessed in the Multi-Ethnic Study of Atherosclerosis (MESA), which showed that compared with White individuals, Black individuals had higher odds of OSA (OR = 1.78), short sleep (OR = 4.95), poor sleep quality (OR = 1.57), and daytime sleepiness (OR = 1.89). Hispanic and Chinese populations also showed higher odds of OSA and short sleep, with Chinese participants having the highest odds of SDB among non-obese individuals [18]. Looking at individual populations, the Hispanic Community Health Study/Study of Latinos (HCHS/SOL) showed that the prevalence of mild, moderate, and severe OSA was 25.8%, 9.8%, and 3.9%, respectively, with higher risk in males (OR = 2.7) [19].

Other research has also confirmed that both OSA and PLMD become more common with advancing age, and that men are generally at higher risk than women for OSA [20]. However, the relationship between these disorders and ethnicity remains less well understood. Emerging evidence suggests that ethnic differences in sleep architecture, access to care, and physiological susceptibility may influence the manifestation and severity of sleep disorders [21]. Studies have shown, for instance, that Hispanic and Black populations may have higher AHI values compared to White populations, even after adjusting for known confounders such as body mass index and comorbidities [22]. Similarly, PLMI has been shown to vary by sex and age, but its association with ethnicity remains underexplored.

Understanding how age, sex, and ethnicity intersect to influence both OSA and PLMD severity is essential for advancing personalized approaches to diagnosis and management. This is especially relevant given the increasing recognition of health disparities in sleep medicine and the need for more inclusive, data-driven frameworks to guide clinical decision-making. For instance, screening for both OSA and PLMSs needs to account for age, sex, and ethnicity, maintaining high clinical suspicion in Black and Hispanic patients. For PLMSs, the observation that older men have higher PLMI highlights the need to consider cardiovascular and cognitive risks in this subgroup. In this study, we conduct a cross-sectional analysis of AHI and PLMI values among a diverse adult patient cohort. We examine differences in these indices across age groups, between sexes, and among ethnic groups. To our knowledge, few studies have concurrently examined both PLMI and AHI across age, sex, and multiple ethnic groups in a large, real-world clinical cohort. Our objective is to identify demographic patterns in the variability of the AHI and PLMI and to determine whether these associations remain significant after adjusting for key covariates. These findings aim to inform future models that incorporate a broader range of clinical and sociodemographic predictors in the assessment and treatment of sleep disorders. Furthermore, integrating these demographic insights into clinical pathways supports more targeted identification, culturally responsive patient education, and individualized treatment strategies, ultimately advancing equity and effectiveness in sleep medicine.

## 2. Materials and Methods

### 2.1. Study Design and Participants

This is a single-center, observational, retrospective study conducted at an academic sleep center in Central California, affiliated with UCSF Fresno. We reviewed consecutive in-lab diagnostic polysomnograms (PSG) studies performed in adults (≥18 years) between June 2022 and July 2024. The exclusion criteria involved studies that were titration, split studies, or incomplete. The PSGs were performed on patients referred from the sleep center and specialty clinics (pulmonary, cardiology, and endocrinology) for evaluation of suspected sleep-disordered breathing or other sleep complaints typical of a tertiary sleep center. The sleep center is an outpatient ambulatory center.

From the PSG report and intake forms, we abstracted age, sex, self-identified ethnicity, and PSG parameters. To preserve de-identification and because of inconsistent documentation across referrals, BMI, medication lists (including dopaminergic agents/antidepressants), iron indices (e.g., ferritin), and detailed comorbidity profiles were not consistently available and therefore not included in the primary analysis (see Sensitivity/limitations below).

The dataset was stratified by age, ethnicity, and sex. Participants self-identified their ethnicity as White, Black, Hispanic, Asian, or Other, consistent with U.S. Census categories. Polysomnographic parameters were analyzed with a particular focus on AHI and PLMI.

The study was conducted in accordance with the Declaration of Helsinki, and approved by the Institutional Review Board (or Ethics Committee) of Community Regional Medical Center (protocol code 2023052 and date of approval of 18 August 2023.

### 2.2. Polysomnography

Polysomnography was performed according to the American Academy of Sleep Medicine (AASM) criteria [23], and data were recorded using the Respironics Alice 6 system. Recorded signals included: EEG (two frontal, two central, two occipital, referenced to contralateral mastoid), EOG, submentalis EMG, bilateral tibialis anterior EMG, airflow (nasal pressure transducer with oronasal thermal sensor), thoracoabdominal effort by inductance plethysmography belts, pulse oximetry, single-lead ECG, synchronized audio and infrared video, microphone for snoring, and body position sensor. Apneas were scored as ≥90% reduction in airflow for ≥10 s; hypopneas as ≥30% reduction in nasal pressure for ≥10 s with ≥3% desaturation and/or arousal, per AASM recommended rule. PLMSs were scored per AASM criteria from tibialis EMG with standard duration and inter-movement interval rules, excluding movements within respiratory event windows per guideline. The study was performed by a registered polysomnographic technologist (RPSGT) and the scoring was verified by a sleep medicine fellow (HM and MP) and by a board-certified sleep physician (LD) according to the AASM criteria [24].

### 2.3. Statistics

All analyses were conducted using STATA 18.0. Covariates included age, sex, and race/ethnicity. Age was analyzed as a continuous variable and also categorized into quartiles for descriptive analyses. Sex was coded as male or female as documented in the medical record. Race/ethnicity was based on patient self-identification at registration and categorized as White, Black, Hispanic, Asian, or Other.

Descriptive analyses were first conducted to summarize the demographic and clinical characteristics of the sample (N = 711). Continuous variables were summarized using means, standard deviations, and interquartile ranges (IQRs), while categorical variables were described using frequencies and percentages. Quantiles of age were constructed, and ANOVAs were used to assess significant bivariate differences between the outcomes of PLMI and AHI by age category, sex, and ethnicity. *p*-values less than 0.05 were considered significant. Of these, significant differences in PLMI were detected in age, sex, and ethnicity. For AHI, significant differences were detected for age and sex.

To determine associations controlling for other covariates, two linear regression models predicting PLMI and AHI were fit, including all three covariates (although here age was kept continuous). 

## 3. Results

### 3.1. Demographics

The sample had a mean age of 57.2 years (SD = 17.9), with 55.6% identifying as female. The majority of participants were White (53.7%), followed by Hispanic (31.6%). Full descriptive statistics are presented in Table 1 and Table 2. In unadjusted analyses, PLMI was significantly higher in older adults, men, and White participants, whereas AHI was higher in men and peaked in the 44–62 year age group (Table 3).

### 3.2. Regression

In multivariable regression models (Table 4), male sex (vs. female) was associated with an increase of 6.0 PLMI/h (*p* < 0.01) and 8.6 AHI (*p* < 0.001). Each additional year of age predicted an increase of 0.37 PLMS/h (*p* < 0.001) but was not significantly related to AHI. Compared with White participants, Black participants had 12.7 fewer PLMS/h (*p* < 0.05), while Hispanic participants had 5 higher AHI events per hour (*p* < 0.05).

### 3.3. Figures

**Figure 1:**
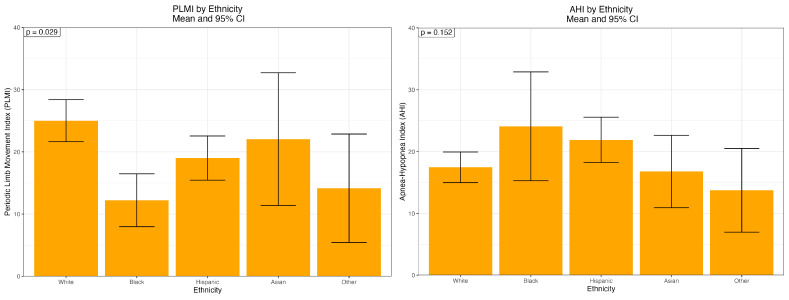
Distribution of PLMI and AHI by ethnicity.

Figure 1 displays mean PLMI values by ethnicity. Error bars represent the standard error of the mean (SE). PLMI was highest among White participants and lowest among Black participants (*p* = 0.029). AHI demonstrated a different pattern with no ethnic differences (*p* = 0.152).

**Figure 2:**
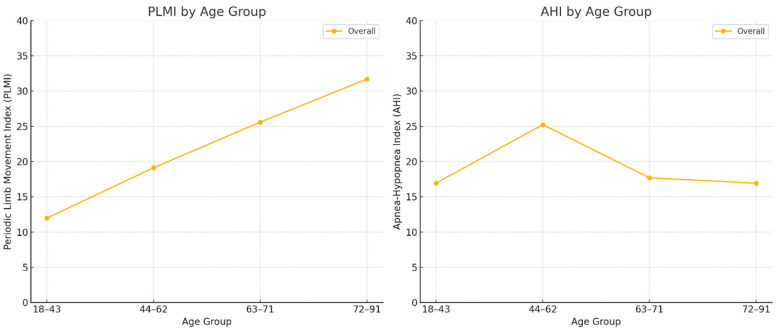
Distribution of PLMI and AHI by combined age group.

Figure 2 displays the distributions of the periodic limb movement index (PLMI) and the apnea–hypopnea index (AHI), in events per hour, stratified by combined age groups. 

## 4. Discussion

In this cross-sectional analysis of a diverse adult cohort, we observed that the PLMI and the AHI varied significantly by age, sex, and ethnicity. PLMI increased steadily with age and was significantly higher in men than women, whereas AHI also showed sex differences but followed a non-linear pattern peaking in middle adulthood and declining slightly in older adults. Ethnic differences were more robust for PLMI with White participants showing the highest and Black participants showing the lowest index.

The age-related rise in PLMI is consistent with prior studies showing that limb movements become more frequent with advancing age, even in individuals without comorbid sleep disorders [25]. This age-related increase may reflect neurobiological changes in dopaminergic pathways that regulate motor activity during sleep [26]. The higher PLMI in men aligns with earlier work linking male sex to increased motor activity during sleep, possibly related to hormonal or neuroanatomical differences [27]. Our findings also reinforce well-established sex differences in OSA [28], with men showing higher AHI than women, a pattern widely reported across sleep epidemiology studies [29]. This disparity has been attributed to anatomical differences in upper airway structure, differences in fat distribution, and hormonal influences [30]. Notably, despite females reporting comparable or greater daytime sleepiness in some studies, their AHI scores are often lower, reflecting potential underestimation of OSA severity when relying solely on respiratory indices [31].

While AHI differences across ethnic groups were not statistically significant in our adjusted models, Hispanic individuals had higher mean AHI values compared to White participants, consistent with prior reports of increased OSA severity among Hispanic adults [18].

From a clinical standpoint, the mean PLMI in our sample was 21.9 (SD = 31.0), already above the diagnostic threshold of 15 events/h for periodic limb movement disorder (PLMD). This emphasizes that PLMSs were not only prevalent but also clinically significant in our cohort. The demographic differences we observed, such as approximately six additional movements per hour in males compared to females, or nearly thirteen fewer movements per hour in Black participants compared to White participants, are large enough to influence diagnostic classification if other symptoms warrant the diagnosis of PLMD. Therefore, even modest group-level differences may translate into clinically meaningful shifts for individual patients, both in terms of diagnosis and in their associated risks for cardiovascular and cognitive comorbidities. While the lower PLMI in Black participants compared to White participants may represent a true biological variation, such as differences in iron metabolism, dopaminergic function, or sleep architecture, it may also suggest that movement disorders manifest differently across racial and ethnic groups. Other neurological movement disorders, such as Tourette syndrome and Huntington’s disease, are known to be underrecognized and underdiagnosed in racial and ethnic minority groups [32]. In sleep medicine, restless legs syndrome has been reported less frequently among Black individuals [33]. Kutner observed a lower prevalence of restless legs syndrome in Black patients undergoing dialysis compared to White patients, which they attributed to higher ferritin levels in the former group [34]. However, when adjusting for socioeconomic factors, Lee et al. found no difference in the prevalence of restless legs syndrome symptoms between Black and White participants [35]. These findings underscore that racial differences in PLMS cannot be interpreted solely as biological. Importantly, current PLMS scoring systems were developed largely in White populations and may underestimate the clinical burden of sleep-related movement disorders in minority groups if movement characteristics differ. Thus, a lower PLMI does not necessarily equate to lower morbidity. For instance, in children there is a significant burden of non-periodic limb movements of sleep, which would not be captured by traditional PLMS scoring metrics [36]. Our findings, therefore, highlight the need to investigate alternative markers of sleep-related movement disorders and to account for structural and cultural factors, including provider recognition, referral pathways, and access to care, that influence how PLMS are measured and diagnosed across diverse populations.

In multivariate models, age predicted higher PLMI but was not significantly associated with AHI, reinforcing the idea that these two sleep disorders have distinct pathophysiology and demographic determinants [37].

The elevated PLMI in older White adults may carry important implications for the assessment of cognitive decline and cardiovascular risk, given prior evidence linking PLMI to hypertension and cognitive decline [25,27,37].

Conversely, the higher AHI observed among Hispanic participants aligns with data from the Multi-Ethnic Study of Atherosclerosis (MESA), reporting greater OSA burden in Hispanic and Black adults [18,22]. These disparities may reflect differences in upper airway anatomy, adiposity distribution, or access to care, highlighting the need for culturally sensitive screening and diagnostic strategies. Hormonal influences may contribute to the sex differences observed, as estrogen and progesterone are known to modulate respiratory drive and upper airway stability [30,38]. Furthermore, the non-linear relationship between age and AHI, particularly the plateau in older adults, may reflect survivor bias or age-related alterations in sleep architecture, as described in earlier community-based cohorts [37]. Future research should explore whether these demographic patterns modify response to treatment or long-term outcomes, especially in underserved populations. Also, future studies may explore other neurophysiological methodologies that may provide additional insight into demographic differences in sleep-related indices. For example, recent studies using EEG markers such as P300 latency and spectral band rhythms have demonstrated their value as objective correlates of cognitive and clinical symptoms across diverse conditions. Fabio et al. showed that electrophysiological parameters can sensitively capture intervention-related changes in Parkinson’s disease [39], while Gangemi et al. reported persistent alterations in P300 and beta rhythms in patients with post-COVID cognitive fog, even months after recovery [40]. Although these studies address different contexts, they underscore how neurophysiological tools complement clinical measures by linking physiological markers to functional outcomes.

This study has several limitations, mainly due to the retrospective design precluding assessment of outcomes or progression of PLMI or AHI. Second, while the cohort was diverse, it was drawn from a single regional academic center, which may limit generalizability to broader populations or geographic areas. An important limitation of this study is the absence of additional covariates such as body mass index, medication use, iron status, and comorbidity profiles. These data could not be consistently included because they were frequently recorded at different time points relative to the diagnostic sleep study, introducing potential misclassification bias. As a result, only age, sex, and ethnicity were analyzed as covariates. Furthermore, because this was a single-center study, the influence of geographic, socioeconomic, and institutional factors on both sample composition and observed outcomes cannot be excluded. Future prospective studies with standardized and contemporaneous collection of anthropometric, biochemical, and clinical data will be needed to strengthen risk adjustment and refine the interpretation of demographic differences.

This study contributes to a growing body of evidence emphasizing the importance of demographic factors in assessing and managing sleep disorders. The clear differences in PLMI and AHI by sex and age, and to a lesser extent by ethnicity, highlight the need for personalized approaches in sleep medicine. Recognizing demographic differences in PLMI and AHI suggests that risk stratification, screening thresholds, and clinical follow-up may need to be adapted based on age, sex, and racial/ethnic background. For example, heightened surveillance for PLMS in older men or for OSA in Hispanic populations may improve early detection. In addition, acknowledging the role of social determinants of health underscores the importance of culturally tailored education, equitable access to diagnostic testing, and individualized treatment strategies. These avenues may enhance both the precision and the equity of care for sleep disorders. Future research should explore how sociodemographic and clinical factors interact to influence symptom severity and treatment response.

## 5. Conclusions

This study highlights significant demographic variations in the severity of PLMS and OSA within a large and ethnically diverse clinical cohort. Our findings reveal that age, sex, and ethnicity are independently associated with the PLMI and AHI, underscoring the need to understand demographics in our approach to sleep disorder assessment and management.

From an epidemiological perspective, this study provides critical evidence for the differential burden of sleep disorders across demographic groups. The observed disparities, such as elevated PLMI in older, White, and male participants and higher AHI in Hispanic individuals, point to underlying biological, behavioral, and social determinants that may shape sleep disorder risk, identification, presentation, and management. These findings support the growing imperative to incorporate sociodemographic factors into predictive models of sleep health and to design interventions that are culturally and clinically tailored.

Given the public health impact of untreated sleep disorders on cardiometabolic outcomes, cognitive function, and quality of life, identifying high-risk populations through epidemiological surveillance is essential. Our study adds to the evidence base informing such efforts and advocates for increased representation of diverse populations in sleep research. Future longitudinal and multi-center studies should explore how these demographic differences translate into disparities in diagnosis, treatment access, and health outcomes over time.

## Figures and Tables

**Table 1:** Descriptive statistics of continuous sleep and demographic variables.

Variable	Mean	Std Dev	IQR
Age (years)	57.17	17.93	(44, 71)
Sleep Latency (min)	30.06	35.66	(8.5, 36.5)
REM Latency (min)	169.22	95.99	(90, 231.05)
Wake after Sleep onset (min)	89.08	84.25	(38.8, 116.5)
Total Sleep Time (min)	298.73	91.70	(248.5, 363.5)
Minimum Oxygen saturation (%)	82.27	6.98	(79, 87)
Mean Oxygen Saturation (%)	93.12	2.32	(92, 95)
Awake percent (%)	28.91	20.16	(13.7, 39)
N1 percent	5.86	4.25	(3.3, 7.2)
N2 percent	44.87	15.76	(35.6, 55.5)
N3 percent	9.35	7.47	(4, 13.2)
REM percent	10.99	7.74	(4.7, 16.2)
Central apnea index (events/h)	1.12	3.65	(0, 0.6)
Total Apnea–Hypopnea index (events/h)	19.29	25.79	(4.5, 22.55)
Periodic Limb Movement Index (PLMS/h)	21.85	31.02	(1.1, 30.2)

**Table 2:** Distribution of categorical demographic and clinical characteristics (N = 711).

	Count	%
**Sex**		
Female	395	55.56%
Male	316	44.44%
**Ethnicity**		
White	380	53.67%
Black	43	6.07%
Hispanic	224	31.64%
Asian	48	6.78%
Other	13	1.84%

**Table 3:** Mean PLMI and AHI by age group, sex, and ethnicity with ANOVA *p*-values.

	PLMI			AHI		
	N	M (SD)	*p*-Value	N	M (SD)	*p*-Value
Age						
18–43	178	11.97 (19.08)	<0.001	178	16.94 (22.02)	0.004
44–62	188	19.11 (29.18)		187	25.23 (33.46)	
63–71	177	25.58 (33.41)		177	17.69 (23.79)	
72–91	166	31.7 (36.88)		165	16.92 (20.24)	
Sex						
Female	395	18.92 (26.7)	0.005	393	15.41 (24.04)	<0.001
Male	315	25.53 (35.41)		315	24.12 (27.09)	
Ethnicity						
White	379	25.02 (33.66)	0.029	379	17.47 (24.6)	0.152
Black	43	12.23 (14.23)		43	24.09 (29.39)	
Hispanic	224	19.01 (27.11)		223	21.9 (27.93)	
Asian	48	22.05 (37.74)		48	16.79 (20.66)	
Other	13	14.15 (16.04)		12	13.76 (12.44)	

**Table 4:** Multivariable linear regression models predicting PLMI and AHI by age, sex, and ethnicity.do.

	PLMI	AHI
	B (SD)	B (SD)
Intercept	−0.02 (4.26)	12.25 (3.59) ***
Age	0.37 (0.06) ***	0.02 (0.05)
Sex (reference Woman)	6.02 (2.29) **	8.55 (1.93) ***
Ethnicity (reference white)		
Black	−12.71 (4.9) *	7.26 (4.12)
Hispanic	−3.04 (2.58)	4.97 (2.17) *
Asian	−0.44 (4.63)	−0.67 (3.9)
Other	−11.28 (8.53)	−1.28 (7.45)

* *p* < 0.05, ** *p* < 0.01, *** *p* < 0.001.

## Data Availability

The data are not publicly available due to institutional regulations.

## Abbreviations

The following abbreviations are used in this manuscript:

AASM	American Academy of Sleep Medicine
AHI	Apnea–Hypopnea Index
EEG	Electroencephalogram
EMG	Electromyogram
IRB	Institutional Review Board
OSA	Obstructive Sleep Apnea
PLMD	Periodic Limb Movement Disorder
PLMI	Periodic Limb Movement Index
SD	Standard Deviation
